# The subjective and objective very long-term outcomes of TVT in the COVID era: A 20-year follow-up

**DOI:** 10.1007/s00192-022-05094-9

**Published:** 2022-03-01

**Authors:** Andrea Braga, Giorgio Caccia, Andrea Papadia, Fabiana Castronovo, Stefano Salvatore, Chiara Scancarello, Marco Torella, Fabio Ghezzi, Maurizio Serati

**Affiliations:** 1 Department of Obstetrics and Gynaecology, EOC - Beata Vergine Hospital, Mendrisio, Switzerland; 2 Faculty of Biomedical Sciences, Università della Svizzera Italiana, Lugano, Switzerland; 3 Department of Obstetrics and Gynaecology, EOC - Civico Hospital, Lugano, Switzerland; 4 Department of Obstetrics and Gynaecology, IRCCS San Raffaele Scientific Institute, Milan, Italy; 5 Department of Obstetrics and Gynaecology, University of Insubria, Varese, Italy; 6 Department of Gyanecology, Obstetric and Reproductive Science, Second University of Naples, Naples, Italy

**Keywords:** COVID, Long-term follow-up, Mid-urethral sling, Stress urinary incontinence, TVT

## Abstract

**Introduction and hypothesis:**

Few studies in literature have assessed the long-term durability and mesh-related complications of mid-urethral slings (MUSs). The aim of this study is to assess the efficacy and safety of retro-pubic tension-free vaginal tape (TVT) 20 years after implantation for the treatment of female stress urinary incontinence (SUI).

**Methods:**

A prospective observational study was conducted in two urogynaecologic units in two countries. All the patients involved were consecutive women with urodynamically proven pure SUI treated by TVT. The patients underwent preoperative clinical and urodynamic evaluations. Subjective outcomes, objective outcomes and adverse events were recorded during the follow-up period.

**Results:**

Fifty-two patients underwent a TVT surgical procedure. Twenty years after surgery, 32 out of 36 patients (88.8%) declared themselves cured (*p* = 0.98). Similarly, 33 out of these 36 patients (91.7%) were objectively cured (*p* = 0.98). No significant deterioration of subjective and objective cure rates was observed over time (*p* for trend 0.50 and 0.48). Fifteen of the 36 patients (41.6%) at the 20-year follow-up reported the onset of de novo overactive bladder (OAB) (*p* = 0.004). No significant vaginal bladder or urethral erosion or de novo dyspareunia was recorded and no patient required tape release or resection during this period. The cause of death of seven out of ten women who died in the last year of the follow-up period was coronavirus disease 19 (COVID 19).

**Conclusions:**

The 20-year results of this study showed that TVT is a highly effective and safe option for the treatment of SUI. The impact of COVID 19 on the mortality rate of elderly women has drastically reduced the number of eligible patients for future evaluations in our region.

## Introduction

Since their introduction [1], tension-free vaginal tape mid-urethral slings (Gynecare TVT System®) have been considered the most effective and safest procedure for the treatment of female stress urinary incontinence [2–4]. However, in recent years, the criticisms regarding the use of transvaginal mesh for pelvic organ prolapse (POP) repair have also involved the mesh intended for the treatment of SUI.

Although the notice issued by the United States Food and Drug Administration (US FDA) considered MUSs to be relatively safe [5], in July 2018 the British government announced that the use of mesh for the treatment of POP and urinary incontinence (UI) was to be paused. Conversely, leading urological and urogynaecological associations have stated the importance of distinguishing between the mesh used for POP and the one used for SUI [6]. In a review of statements on the use of synthetic mesh for POP and SUI, the Urogynaecology and Pelvic Floor Committee within the International Federation of Gynaecology and Obstetrics (FIGO) drew the conclusion that there is strong evidence to support the use of synthetic mesh MUSs in the treatment of SUI [7]. In fact, in short- and medium-term follow-ups, a large amount of published data has confirmed the similar efficacy but less morbidity of synthetic slings compared with conventional non-mesh sling techniques [8, 9]. Only few studies in literature have assessed the long-term durability and the mesh-related complications of MUSs, producing results that are extremely encouraging [2, 10, 11]. Nevertheless, there is not enough evidence available on the long-lasting benefits of MUS procedures to be able to recommend these techniques with a robust level of evidence.

Ideally, randomized or prospective clinical trials with a long-term follow-up would be the optimal method for evaluating sling procedures. However, this type of study represents a great challenge for researchers due to many factors that could influence the results, such as cost, the time required and the bias that may occur, especially if there is a significant loss of subjects during the follow-up period (withdrawal by the subjects or death). The latter point is crucial for the long-time evaluation of any surgical procedure. Unfortunately, in the last year, many countries have reported an increased mortality rate due to the novel severe acute respiratory syndrome coronavirus 2 (SARS-CoV-2).

The aim of the present study is to evaluate the long-term efficacy and safety of a TVT procedure in women who underwent a minimum 20-year follow-up.

## Materials and methods

This is an update of a previous prospective study performed in two urogynaecologic units, namely the EOC-Beata Vergine Hospital, Mendrisio, Switzerland, and the University of Insubria, Varese, Italy. As reported in the previous study [2], we enrolled all consecutive women who complained of pure SUI symptoms with urodynamically proven USI between January 1998 and January 2000. All patients deemed eligible for surgical treatment were scheduled for a TVT procedure (Gynecare TVT System®; Ethicon Inc., Somerville, NJ, USA). Women with a previous history of radical pelvic surgery, psychiatric and neurologic disorders, concomitant vaginal prolapse > stage 1 according to the Pelvic Organ Prolapse Quantification (POP-Q) system [12], OAB symptoms, urodynamically proven DO and postvoid residual > 100 ml were excluded from the study. The preoperative evaluation included the patient’s medical history, a physical examination, a voiding diary, urinalysis and complete urodynamic testing. A physical examination was performed with the patient in the lithotomy position and POP was described during a maximal Valsalva manouevre according to the POP-Q system [12]. All the women were evaluated with urodynamic studies as previously described [13] [including uroflowmetry, filling cystometry, Valsalva leak-point pressure (VLPP) measurement and a pressure/flow study] by a trained urogynaecologist, using a standardized protocol in accordance with the best urodynamic practice guidelines of the International Continence Society [14]. Urethral hypermobility was defined by a Q-tip test > 30°. Patients were included regardless of their Q-tip test and VLPP values. All methods, definitions and units were updated in agreement with the latest version of the International Continence Society standardization of terminology [15]. All patients also completed the International Consultation on Incontinence Questionnaire-Short Form ICIQ-SF [16]. All the TVT procedures were performed according to the technique originally described by Ulmsten [1]. General or spinal anaesthesia was used in accordance with anaesthesiological requirements and/or the patient’s preference, as previously reported [17]. Postoperative evaluations were mandatory at 1, 5, 10, 15, 17 and 20 years in the two centres and intermediate visits were scheduled at the physician’s discretion. The standard of care follow-up in our clinics is that all subjects are asked to complete the following questionnaires and undergo the physical examination components listed in the follow-up evaluation. Every follow-up visit included the patient’s medical history, a physical examination, a voiding diary, a stress test and an evaluation of subjective satisfaction [18]. A stress test was performed in the lithotomy and upright positions with a full bladder (ultrasonographic measurement > 400 ml). “Objective cure” was defined as the absence of urine leakage during the stress test. To define the subjective outcomes at 1, 5, 10, 15, 17 and 20 years, all patients completed the ICIQ-SF, the PGI-I scale (a 7-point scale, with a range of responses from 1, “very much improved”, through to 7, “very much worse”) [19] and a patient-satisfaction scale (a single, self-answered, Likert-type scale of 0–10 that grades the patient’s degree of satisfaction regarding continence, where 0 represents “not satisfied,” and 10, “satisfied”) [20]. Subjective success was indicated by both “very much improved” or “much improved” (PGI-I ≤ 2) and a patient-satisfaction score ≥ 8, as previously described in 2011 by Abdel-Fattah et al. [21]. Additional UDSs were also performed when women complained of the onset of de novo overactive bladder (OAB) symptoms. The Declaration of Helsinki was followed, and preoperative written informed consent for TVT implantation was obtained from all the patients in this observational prospective evaluation. The study does not require ethics/institutional review board approval because normal clinical practice has been followed [22].

## Statistical analysis

Statistical analysis was performed with IBM-SPSS v.17 for Windows (IBM Corp, Armonk, NY, USA). Continuous variables were reported as the median and interquartile range. We used the χ^2^ test and χ^2^ test for trend to analyse and compare the surgical outcomes during follow-up. The χ^2^ test for trend can better assess if the success of the surgical procedure tends to decrease over time by comparing the cure rates at the different follow-up visits (1, 5, 10, 15, 17 and 20 years). The null hypothesis is that there is no association between the cure rate of TVT and the time. The one-way analysis of variance was used to compare continuous series of variables in the comparison of the scores used to measure the subjective outcomes. The Cox proportional hazards model was used for univariate analysis to evaluate factors potentially affecting the risk of recurrence (subjective or objective) during the study period. Statistical significance was considered achieved when *p* < 0.05. The analysis of success data was performed by plotting by Kaplan-Meier survival curves, which were compared using the long-rank (Mantel-Cox) test.

## Results

Fifty-two consecutive women with proven USI, who fulfilled the inclusion criteria, i.e., had undergone a TVT procedure, were considered. At the 20-year follow-up, 36 (69.2%) patients were available for evaluation. The baseline patient characteristics of these 36 patients are reported in Table 1. At the 20-year follow-up, six patients (11.5%) were lost to follow-up or could no longer be evaluated, while ten (19.2%) had died from medical causes unrelated to the TVT procedure (2 after the 10-year examination, 1 after the 15-year examination and 7 after the 17-year examination).

**Table 1:** Baseline patients characteristics

Characteristics	*n* = 52
Age, years, median, (IQR)	60 (51–72)
BMI, kg/m², median, (IQR)	25.9 (25–28)
Obese, BMI ≥ 30, no. (%)	6 (11.5)
Sexually active, no. (%)	40 (77)
Menopausal, no. (%)	43 (82.3)
HRT, no. (%)	17 (32.7)
Recurrent UTI, no. (%)	0 (0)
Previous vaginal deliveries, median, (IQR)	2 (1–4)
Macrosome (≥ 4000 g), no. (%)	5 (9.6)
Operative delivery (vacuum/forceps), no. (%)	4 (15.4)
Previous hysterectomy, no. (%)	12 (46.1)
Urethral hypermobility, no. (%)	44 (84.6)
VLPP < 60 cmH_2_O, no. (%)	28 (53.8)
ICQ-SF preop.	17 (16–17)

IQR: interquartile range; BMI: body mass index

HRT: hormone replacement therapy; UTI: urinary tract infection

**Fig. 1:**
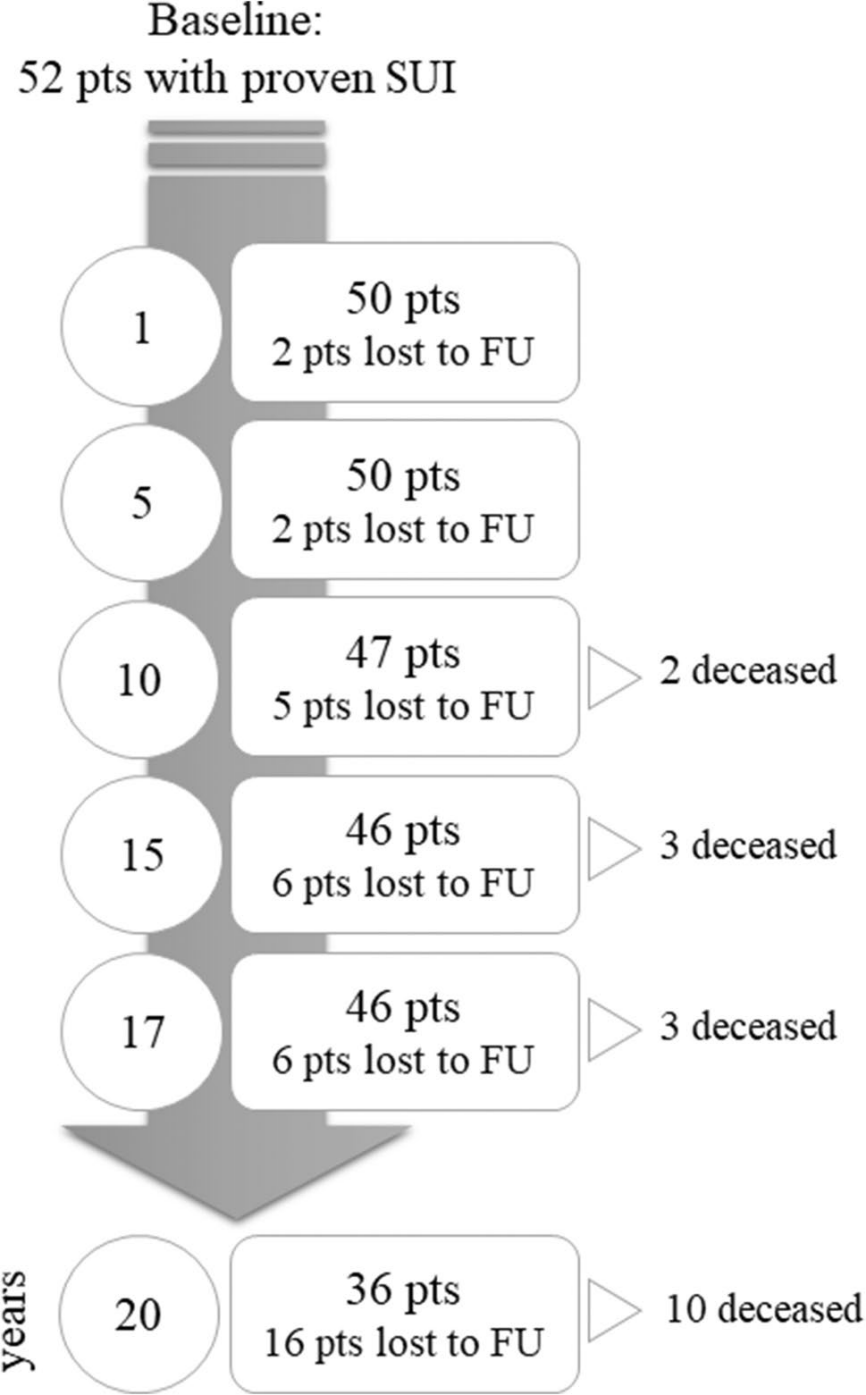
Flow chart TVT 20 years

**Fig. 2:**
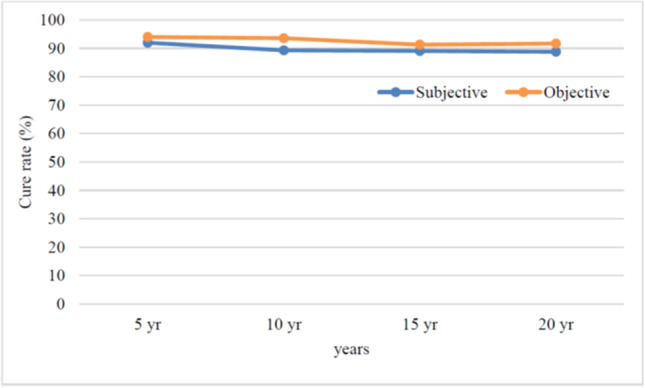
Recurrence-free (disease-specific) survival curve of women undergoing TVT

Figure 1 displays the flowchart of the study. All the women lost to follow-up were subjectively cured at their last follow-up visit. Subjective and objective cure rates are summarized in Table 2 and displayed in Fig. 2.

**Table 2:** Cure rates at the 1-, 5-, 10-, 15-, 17- and 20-year follow-up visits

	1 yr	5 yr	10 yr	15 yr	17 yr	20 yr	*p* value
Subjective outcomes Satisfied (N)	92% (46/50)	92% (46/50)	89.3% (42/47)	89.1% (41/46)	89.1% (41/46)	88.8% (32/36)	0.98 ^a^ 0.50 ^b^
Objectively cured Objectively cured (at stress test)	94% (47/50)	94% (47/50)	93.6% (44/47)	91.3% (42/46)	91.3% (42/46)	91.7% (33/36)	0.98 ^a^ 0.48 ^b^
De novo overactive bladder Onset of OAB	12% (6/50)	12% (6/50)	19.1% (9/47)	23.9% (11/46)	32.6% (15/46)	41.6% (15/36)	0.004 ^a^

^a^Chi-square test; ^b^chi square test for trend

The null hypothesis is that there is no association between the cure rate of TVT and the time

Twenty years after the TVT procedure, 32 out of the 36 (88.8%) available patients were subjectively cured (*p* for trend 0.50), whereas 33 out of the 36 (91.7%) patients were objectively cured (*p* for trend 0.48). In addition, at the 20-year follow-up, we observed a significant trend of de novo overactive bladder occurrence in 15 out of the 36 patients [(41.6%) (*p* = 0.004)]. The long-term subjective outcomes, reported in Table 3, showed no significant deterioration over time.

**Table 3:** Subjective outcome scores over time after TVT

	Baseline	1 year	5 years	10 years	15 years	17 years	20 years	*p* value
ICIQ-sf	17 (16–17)	0 (0–8)	0 (0–10)	0 (0–10)	0(0–12)	0(0–12)	0(0–12)	< 0.0001^*^
“Very much better” or “much better” on PGI-I		46/50 (92%)		42/47 (89.3%)	41/46 89.1%	41/46 89.1%	32/36 88.8%	
Patient Satisfaction Scale			10 (8–10)		10 (8–10)	10 (8–10)	10 (7–10)	

Data are expressed as an absolute number (%) or median (interquartile range)

*One-way analysis of variance (ANOVA)

The Clavien-Dindo classification of long-term complications is reported in Table 4. No significant vaginal bladder or urethral erosion or de novo dyspareunia was registered in our study population, and no patient required tape release or resection during these 20 years. The univariate analysis did not find any risk factor statistically associated with the recurrence of SUI.

**Table 4:** Clavien**-**Dindo classification of long-term complications

Complication	*N* = 36	Action
Clavien I		
Persistence of voiding dysfunction	2 (3.8%)	Observation
Clavien II		
De novo OAB	15 (41.6%)	Antimuscarinics/β-agonists
*Recurrent UTIs*	2 (3.8%)	Antimicrobial prophylaxis or therapy

Data are expressed as an absolute number (%)

**Fig. 3:**
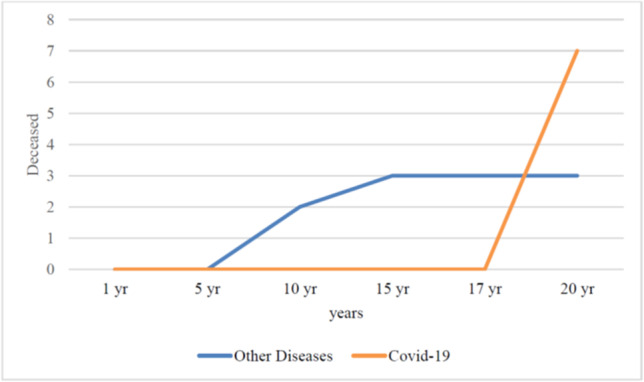
Patients who died during the follow-up period

Interestingly, the cause of death of seven out of the ten women who died in the last year of the follow-up period was COVID 19 (Fig. 3 and Table 5).

**Table 5:** Rate of patients who died and median age

	1 year	5 years	10 years	15 years	17 years	20 years	*p* value
Patients deceased	- -	- -	2 (4.2%)	3 (6.5%)	3 (6.5%)	10 (27.7%)	0.006 ^a^
Median age of pts deceased	–	–	66 (52–81)	71 (52–81)	71 (52–81)	80 (52–95)	0.01*

Data are expressed as an absolute number (%) or median (interquartile range)

^a^Chi-square test

*One-way analysis of variance (ANOVA)

## Discussion

The present study, which is the first in the literature to the best of our knowledge, shows the subjective and objective outcomes of the TVT procedures undergone by women with pure stress incontinence 20 years after surgery. We found that TVT is a highly effective and safe procedure, with very long-lasting efficacy.

Following the notice issued by the US FDA in 2011 and the recent announcement of the British government, the use of MUSs for female SUI has come under scrutiny because of the reports of severe vaginal mesh complications. However, all these restrictions failed to make the distinction between mesh for incontinence and mesh for prolapse. The European Commission’s Scientific Committee on Emerging and Newly Identified Health Risks (SCENIHR) [23] stated that morbidity related to use of prosthetic transvaginal mesh is greater in POP than in SUI treatment.

In fact, as shown by the results of a large population-based cohort study [24], MUS procedures have a lower risk of immediate (adjusted relative risk [aRR] 0.44 [95% CI 0.36–0.55]) and later complications (1.12 [0.98–1.27]) (all ratios are for retropubic mesh) than POP procedures. In particular, compared with non-mesh repair, the mesh repair of anterior compartment prolapse was associated with a significantly increased risk of later complications [3.15 (2.46–4.04)]. This study, which is included in the Scottish Independent Review [25] on the use of transvaginal mesh implants in the treatment of female SUI, supports the use of mesh procedures for incontinence, although further research on longer term outcomes would be beneficial. Moreover, an updated meta-analysis by Fusco et al. [8] confirms the superiority of MUSs over Burch colposuspension in terms of overall and objective continence rates as well as equivalent overall and subjective continence rates regarding pubovaginal slings, although MUSs present a statistically significantly lower incidence of storage lower urinary tract symptoms (LUTS).

Despite these advantages and the low complication rate of MUSs [26], the occurrences of mesh erosion and pain have recently become a major concern. The Cochrane Review Collaboration on the long-term outcomes of MUSs reported low incidence rates of vaginal tape erosion (0.4%–1.5%) and groin pain (0.4%–1.6%) [27]. Another recent study on the long-term safety of sling mesh implants for SUI reported a risk of mesh erosion of 3.7% with a 6.7% reoperation rate at the 7-year follow-up [28]. Nevertheless, the growing international controversy on the use of vaginal mesh has led to these surgical techniques being reconsidered and it has highlighted the need for more information on the long-term results and adverse effects of the mesh used to treat SUI [29]. Unfortunately, two problems arise in this regard:

1. Although the data are encouraging, only two studies in literature have evaluated the long-term outcomes of TVT with a follow-up of at least 17 years. Nilsson et al. [10] reported an objective cure rate of 91.3%, assessed by a cough stress test, and an 87.2% subjective cure rate, evaluated by PGI-I, as well as by some specifically validated questionnaires. However, only 51.1% of the women were assessed with an objective examination, and no UDS evaluation was performed, while in our previous evaluation of the present series [2], 17 years after surgery 89.1% of the women declared themselves cured and 91.4% were objectively cured.
2. The COVID 19 pandemic may represent a significant hurdle for future research on the long-term efficacy and safety of MUSs, especially in regions that have experienced a similar mortality rate to ours.

If it is considered that the mean age of patients dying from SARS-CoV-2 infection is 81 (median 83, range 0–109, IQR 75–88) years and 49.3% of these are women [11], this could limit the long-term evaluation of this technique, especially considering that the median age of patients who underwent a TVT procedure 20 years ago was around 60 years old [2, 3, 10]. This means that we may not have sufficient data available in the long term for an adequate evaluation of this procedure. In the present study we have recorded a great increase in mortality in the 3 years since the last follow-up due to SARS-CoV-2 infection (7/10 overall deaths) (Fig. 3).

It is interesting to note that no late mesh-related complications appeared during the 20-year follow-up period. However, as in the previous study, we recorded a statistically significant growing trend of de novo OAB, which increased from 32.6% to 41.8% in the last 3-year postoperative period.

This observation could reflect the ageing of the patients rather than being a direct consequence of surgery. However, is important to provide adequate preoperative counselling, since anti-muscarinic therapy is less effective in women with de novo overactive bladder after mid-urethral sling placement [30].

The present study has several strengths including: (1) a clinical evaluation performed in all patients 20 years after TVT; (2) a highly homogeneous study population with the exclusion of women with mixed incontinence, DO and/or any other associated surgical procedure; (3) the subjective and objective outcomes available for the 1-, 5-, 10-, 15-, 17- and 20-year postoperative period. We acknowledge that the weaknesses of this study could be the limited sample size and the lack of data of pressure/flow study in all women with de novo OAB. However, we also emphasize that no larger investigations with a similar follow-up, evaluating both objective and subjective outcomes, are available in literature.

## Conclusion

The present study, which is the first reporting a 20-year follow-up, appears to demonstrate that TVT is associated with an excellent long-lasting objective and subjective cure rate, with a negligible rate of mesh-related complications. The dramatic impact of COVID 19 on the mortality rate of elderly women in the last 16 months has drastically reduced the number of eligible patients for future evaluations in our region.
